# Autophagy Is Indispensable for the Self-Renewal and Quiescence of Ovarian Cancer Spheroid Cells with Stem Cell-Like Properties

**DOI:** 10.1155/2018/7010472

**Published:** 2018-09-17

**Authors:** Qian Wang, Shixia Bu, Dedong Xin, Boning Li, Lan Wang, Dongmei Lai

**Affiliations:** ^1^International Peace Maternity and Child Health Hospital, School of Medicine, Shanghai Jiao Tong University, Shanghai 200030, China; ^2^Institute of Embryo-Fetal Original Adult Disease Affiliated to Shanghai Jiao Tong University School of Medicine, Shanghai 200030, China; ^3^College of Chemistry and Life Science, Zhejiang Normal University, Jinhua 321004, China; ^4^Shanghai Center for Systems Biomedicine (Ministry of Education), Shanghai Jiao Tong University, Shanghai 200240, China

## Abstract

Epithelial ovarian cancer has the highest mortality rate of all gynecologic cancers. Cancer stem cells are considered to be the initiating cells of tumors. It is known that spheroid culture promotes ovarian cancer cells to acquire stem cell characteristics and to become stem cell-like. But the mechanisms remain largely unclear. Our data show that autophagy is sustainably activated in ovarian cancer spheroid cells. Inhibition of autophagy by knockdown of ATG5 abolishes the self-renewal ability of ovarian cancer spheroid cells. Knockdown of ATG5 prevents ovarian cancer spheroid cells to enter quiescent state. Autophagy is critical for quiescent ovarian cancer spheroid cells to reenter the cell cycle because rapamycin can promote quiescent ovarian cancer spheroid cells to form colonies on soft agar and knockdown of ATG5 can arrest ovarian cancer cells in G0/G1. Autophagy and NRF2 form a positive feedback regulation loop to regulate reactive oxygen species (ROS) levels in ovarian cancer spheroid cells. The optimal ROS level, neither too high nor too low, facilitates the self-renewal marker, NOTCH1, to reach to the highest level. Bafilomycin A1 can impair the self-renewal of ovarian cancer spheroid cells by disturbing ROS levels.

## 1. Introduction

Epithelial ovarian cancer has the highest mortality rate of all gynecologic cancers. The poor prognosis of ovarian cancer patients was shown by 75% of ovarian cancer patients detected with metastasis [1]. Cancer stem cells are defined as a distinct and small subset of tumor-initiating cells with self-renewal potential that drives the growth and progression of tumors [2, 3]. Ovarian cancer stem-like and initiating cells can be isolated in ovarian cancer cell lines, primary ovarian tumors, and the ascites of ovarian cancer patients. These cells express stem cell markers, resist to chemotherapeutic agents, and establish tumors in animal models [4–6]. It is widely accepted that spheroid culture promotes ovarian cancer cells to acquire stem cell characteristics and become stem cell-like [7, 8]. However, the regulatory mechanisms of ovarian cancer spheroid cells maintaining their stem cell-like properties are largely unknown.

Autophagy is a degradation pathway by which cytoplasmic organelles or cytosolic components are sequestered in a double-membrane-bound autophagosome and delivered to the lysosome for degradation [9]. Autophagy favors tumor progression by helping tumor cells to cope with intracellular and environmental stress, including oxidative stress caused by excessive production of reactive oxygen species (ROS) [10, 11], which are involved in maintaining proper function of stem cells and cancer stem cells [12–14]. Several groups have proposed that autophagy plays important roles in ovarian cancer survival [15], drug resistance [16], and metastasis from different angles [17]. Recently, autophagy has been reported to be able to regulate the self-renewal of ovarian cancer stem cells through regulating FOXA2 [18] and favor chemoresistance of ovarian cancer stem cells [19]. Here, we show that not only the self-renewal ability but also the quiescence maintenance of ovarian cancer spheroid cells can be regulated by autophagy. Autophagy may regulate the self-renewal of ovarian cancer spheroid cells through regulating ROS levels.

## 2. Materials and Methods

### 2.1. Cell Culture

HEK 293T, SKOV3, and HO8910 cell lines were obtained from Shanghai Cell Bank of Chinese Academy of Sciences. The A2780 cell line was a kind gift from Dr. Shu Zhang at Shanghai Renji Hospital (China). Human primary ovarian cancer cells were isolated from human ovarian cancer tissue of a patient hospitalized at the International Peace Maternity and Child Health Hospital. The patient had given informed consent to use the tissue. The clinical sample was approved by the Institutional Review Boards at Shanghai Jiao Tong University, Shanghai, China. The isolation method was reported previously [20]. In brief, 1 cm of tissue was cut into pieces and suspended in 10 ml 0.25% trypsin-EDTA. The solution was filtered and incubated for 15 min in a water bath set at 37°C. The cells were collected by neutralizing the trypsin and by centrifugation. HEK 293T cells and the ovarian cancer adherent cells were cultured in DMEM culture medium containing 10% fetal bovine serum. The ovarian cancer spheroid cells were cultured in DMEM/F12 culture medium containing 10% knockout serum replacement (Gibco), 10 ng/ml bFGF, 10 ng/ml EGF, nonessential amino acids, and sodium pyruvate. All cells were cultured at 37°C in a humidified atmosphere containing 5% carbon dioxide.

### 2.2. Microarray Analysis

RNA samples from SKOV3 adherent and spheroid cells were prepared for the microarray analysis. The microarray was performed by Shanghai Biochip Co., Ltd. (China) with Affymetrix GeneChip arrays. The gene expression data were provided as supplementary material. The heat map of gene differentiation was generated using the method described by Rubin et al. [21]. Briefly, a heat map of the median-centered FPKM (fragments per kilobase of exon per million fragments mapped) values corresponding to the transcripts in samples was generated using the heatmap.2 function of the gplots R package.

### 2.3. Reverse Transcription and q-PCR

Cells were lysed with RNAiso reagent (TaKaRa, Japan). Total RNA was extracted, and 1 *μ*g of RNA was reverse-transcribed with M-MLV Reverse Transcriptase (Promega, USA) and subjected to quantitative PCR (q-PCR). The primer sequences are listed as follows: p62/SQSTM1 forward 5′-CTGAAGAACGTTGGGGAGAG-3′, reverse 5′-GCTCTTCTCCTCTGTGCTGG-3′; NRF2 forward 5′-ATCATGATGGACTTGGAGCTG-3′, reverse5′-GCTCATACTCTTTCCGTCGC-3′; and 18s RNA forward 5′-CGTTGATTAAGTCCCTGCCCTT-3′, reverse 5′-TCAAGTTCGACCGTCTTCTCAG-3′.

### 2.4. Plasmids, siRNA, and shRNA

The tfLC3 plasmid is a kind gift from Dr. Junlin Guan at Cincinnati University (USA). The pLVX-mCherry-C1-Fluc plasmid was purchased from Biogot Technology Co. (China). pMD2.G and psPAX2 plasmids were kind gifts from Dr. Weidong Le at Shanghai Institutes for Biological Sciences, Chinese Academy of Sciences. The siRNA or shRNA for ATG5, negative control (Nc), and NRF2 were reported previously [22–24] and purchased from Shanghai GenePharma Co. (China). For establishing shRNA cell lines, A2780 cells were transfected with pGPU6/shRNA constructs and selected by complete medium containing 100 *μ*g/ml neomycin. For establishing A2780 stably expressing mCherry and luciferase (A2780-mCherry-Luc), HEK 293T cells were transfected with pLVX-mCherry-C1-Fluc, pMD2.G, and psPAX2 to produce lentivirus to transduce A2780 cells in the presence of polybrene. The transduced A2780 cells were selected by complete medium containing 10 *μ*g/ml puromycin.

### 2.5. GSH/GSSG Measurement

Cellular GSH and GSSG were measured with the GSH and GSSG Assay Kit (Beyotime, China) according to the manufacturer's instructions. The OD value was read at wavelength of 490 nM with a BioTek ELISA reader.

### 2.6. Chemicals and Antibodies

H2DCFDA, rapamycin, pyronin Y, and buthionine sulfoximine were obtained from Sigma-Aldrich Co. (USA). Bafilomycin A1 and pepstatin A were obtained from Sangon Biotech Co. (China). D-Luciferin was obtained from Goldbio Co. (USA). Polybrene, Hoechst 33342, and propidium iodide (PI) were obtained from Yeasen Biotech Co. (China). Transfection reagents DharmaFECT, Lipofectamine 2000, and Alexa Fluor 488 and 567 secondary antibodies were obtained from Thermo Fisher Scientific Inc. (USA, 1:500). Anti-LC3 (#3868, 1:1000), anti-Beclin (#3495, 1:1000), anti-ATG5 (#12994, 1:1000), anti-Oct-4 (#2750, 1:1000), and anti-p62/SQSTM1 (#7695, 1:1000) antibodies were obtained from Cell Signaling Technology (USA). Anti-RB1CC1 (sc-22709, 1:100), anti-Ki-67 (sc-23900, 1:100), and HRP-conjugated goat anti-rabbit secondary antibodies were obtained from Santa Cruz Biotechnology (USA). Anti-vimentin, HRP-conjugated goat anti-mouse, and rabbit anti-goat secondary antibodies were obtained from Boster Co. (China). Anti-*β*-tubulin (1:1000) and anti-*β*-actin (1:1000) antibodies were obtained from Transgene Biotech Co. (China). Anti-NRF2 (WL02135, 1:2000), anti-NOTCH1 (WL01991, 1:500), and anti-Lamin B (WL01775, 1:500) antibodies were obtained from Wanleibio Co. (China).

### 2.7. Western Blot and Immunofluorescence

For Western blot analysis, cells were lysed in RIPA buffer and quantified for protein concentration. Next, 20 *μ*g of protein was loaded onto the gel and subjected to Western blot. Western blot results were quantified by ImageJ (NIH) software. For immunofluorescence, cells were fixed with 4% paraformaldehyde, stained with primary and fluorescent secondary antibodies. Images of cells were taken using a Leica fluorescence microscope or a Leica SPE confocal laser scanning microscope system.

### 2.8. ROS Analysis

Cells were stained with 20 *μ*M H2DCFDA in DMEM without serum at 37°C for 15 min, washed with PBS, fixed in 4% paraformaldehyde, and subjected to flow cytometric analysis (Beckman Coulter or BD FACS).

### 2.9. Analyses of Cell Cycle and G0 Cell Population

For cell cycle analysis, cells were fixed in 75% ethanol at −20°C overnight, digested with 0.1 mg/ml RNase A at 37°C for 30 min, stained with 40 *μ*g/ml propidium iodide, and subjected to flow cytometric analysis. For G0 cell population analysis, we followed Kim et al.'s protocol [25] with some modifications. Briefly, cells were collected, washed with PBS, fixed in 75% ethanol at −20°C overnight, and incubated with anti-Ki-67 antibody at 37°C for 1 h and secondary antibody at room temperature for 30 min. Cells were then digested with RNase A and stained with propidium iodide and subjected to analysis with their cell cycle and Ki-67 intensity by a flow cytometer (Beckman Coulter or BD FACS).

### 2.10. PY^Low^ and PY^High^ Cell Sorting

PY^Low^ and PY^High^ cell sorting followed Gothot et al.'s protocol [26] with some modifications. Briefly, cells were cultured for 24 h, trypsinized and stained with Hoechst 33342 (5 *μ*g/ml) in Hst buffer at 37°C for 1 h, and continued to be cultured in suspension for another 24 h. Hst buffer consisted of glucose-free medium (#11966025, Gibco) and 2% fetal bovine serum. Cells were stained with Hst again before sorting, followed by pyronin Y staining (1 ng/ml) at 37°C for 30 min. The cells were sorted on a BD FACS.

### 2.11. Soft Agar Assay

Cells suspended with 0.3% soft agar were seeded on top of the lower layer (0.6% agar). The feeder layer of 0.3% agar was replenished every 3 d to prevent desiccation. After 14 or 28 d, the numbers and diameters of colonies were counted and measured either with a microscope or with a gel imaging system (Tanon).

### 2.12. Human Tumor Xenografts

Female BALB/c nude mice (4 weeks old) were obtained from Shanghai Jiao Tong University School of Medicine (Shanghai, China). All experimental protocols were approved by the Ethics Committee of the School of Medicine of Shanghai Jiao Tong University, which were in accordance with the approved guidelines set by the Institutional Animal Care and Use Committee. The mice were intraperitoneally (ip) injected with 150 *μ*l phosphate-buffered saline (PBS) containing 2 × 10^6^ A2780 spheroid cells. After 1 week, the mice were ip injected with DMSO or bafilomycin A1 (0.1 mg/kg) for twice with 3 days of interval. The mice were sacrificed by cervical dislocation under anesthetic status after 28 days, and the tumor xenografts were collected. For animal imaging assay, the mice were ip injected with 2 × 10^6^ A2780-mCherry-Luc spheroid cells. After 1 week, the mice were ip injected with DMSO or bafilomycin A1 (0.1 mg/kg) twice with 3 days of interval. After 28 days, the mice were ip injected with D-luciferin (15 mg/kg) and scanned with imaging system (Roper Scientific).

### 2.13. Statistical Analysis

Student's *t*-test was used to analyze the data. *P* values were calculated in individual assays, and *P* < 0.05 was considered as statistically significant.

## 3. Results

### 3.1. Spheroid Culture Induces Autophagy in Ovarian Cancer Cells

**Figure 1:**
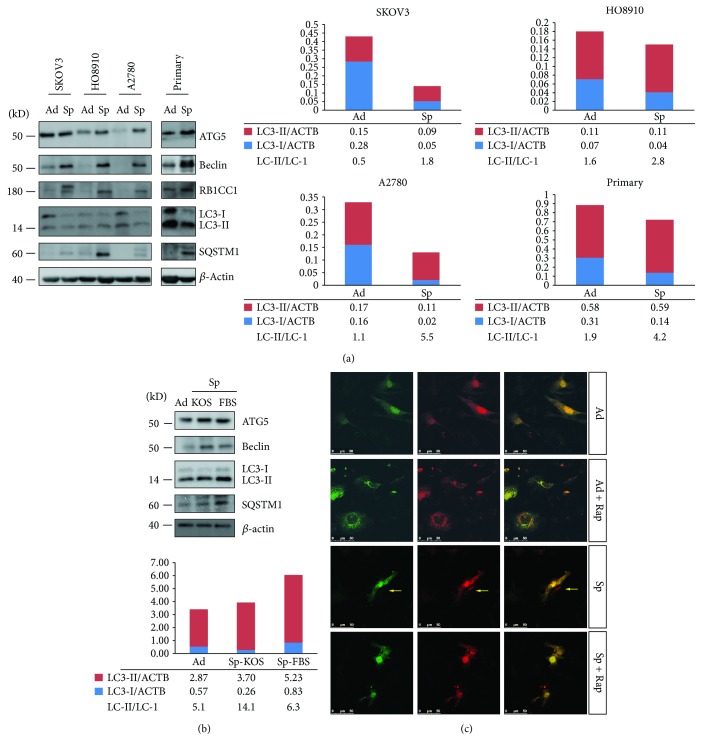
Autophagy is activated in ovarian cancer cells under spheroid culture condition. (a) Western blot analysis of autophagy essential genes and markers in ovarian cancer adherent and spheroid cells. Three ovarian cancer cell lines, SKOV3, HO8910, and A2780, and one primary ovarian cancer cell strain were used. Cells were cultured under adherent or spheroid condition for 48 h and collected for Western blot analysis (adherent (Ad), spheroid (Sp)). Western blot results were quantified by ImageJ (NIH) software. The relative intensity of LC3-I or LC3-II normalized to *β*-actin (ACTB) was shown. The ratios of LC3-II/LC3-I were calculated. (b) Western blot analysis of autophagy essential genes and markers in A2780 spheroid cells in media with either knockout serum replacement (KOS) or fetal bovine serum (FBS) as supplements. The quantification method was described in (a). (c) Autophagy activity analysis in SKOV3 adherent or spheroid cell with tfLC3. SKOV3 cells were transiently transfected with tfLC3 plasmid for 24 h, trypsinized, and cultured under adherent or spheroid conditions for another 48 h with or without rapamycin (Rap, 10 *μ*M). Fresh media with or without Rap were changed every 24 h. The spheroid cells were trypsinized and seeded back to attach to the plate for 4 h before observation. The fluorescent images were scanned with a Leica confocal system.

The ovarian cancer cells can form spheroid cells under anchorage independent conditions in the absence of extracellular matrix attachment. Four ovarian cancer cell strains were used to analyze the difference between ovarian cancer adherent and spheroid cells. The morphology of SKOV3, HO8910, and A2780 adherent and spheroid cells is shown in Figure S1. One primary ovarian cancer cell strain was isolated from ovarian cancer tissue [20]. Epithelial cells and fibroblasts were the two major populations derived from primary ovarian cancer tissue, which can be differentiated by keratin 18 stain. The keratin 18-positive epithelial cells can form spheroid cells (Figures S2(a) and S2(b)). cDNA array data showed that several autophagy pathway essential genes, including MAP1LC3B, ATG16L1, RB1CC1, and ULK1, were upregulated in SKOV3 spheroid cells compared with adherent cells (Figure S3(a)), suggesting that autophagy might be activated in SKOV3 spheroid cells. Western blot analysis showed that the protein levels of RB1CC1 and Beclin were higher in spheroid cells of all four cell strains compared with adherent cells (Figure 1(a)). LC3-II/LC3-I ratios were higher in spheroid cells compared with adherent cells (Figure 1(a)) and can be decreased by autophagy inhibitors bafilomycin A1 or chloroquine (Figure S3(b)), confirming that autophagy was activated in ovarian cancer spheroid cells. To study whether the different autophagy fluxes between adherent and spheroid cells was caused by the different culture media, the cells were grown under spheroid culture conditions in media suitable for stem cells (KOS) or differentiated cells (FBS) and analyzed with Western blot. As shown in Figure 1(b), ATG5, Beclin, and LC3-II/LC3-I ratio increased in spheroid cells cultured in either media compared with adherent cells. However, the LC3-II/LC3-I ratio was lower in the FBS group compared with the KOS group. These results suggested that anchorage independent culture condition and media were the major and minor contributing factors for autophagy activation. Our results were consistent with the previous reports that extracellular matrix detachment can induce autophagy [27, 28].

We observed that the higher LC3-II/LC3-I ratios in ovarian cancer spheroid cells compared with adherent cells were caused by reduced LC3-I levels rather than increased LC3-II levels (Figure 1(a)). This result suggested that autophagy might have been activated for a long time in spheroid cells because sustained autophagy activation leads to degradation of LC3-II in the autolysosome [29]. To further confirm whether autophagy was sustained activated, we investigated the dynamics of autophagosome formation and maturation with tandem mRFP-GFP-LC3 (tfLC3), by which autophagosomes marked both mRFP and GFP signals, while autolysosomes marked only mRFP signals [30]. As shown in Figure 1(c), SKOV3 adherent cells did not show any puncta, which could be stimulated with both green puncta and red puncta by autophagy inducer rapamycin. SKOV3 spheroid cells showed a lot of red puncta and much less green puncta with very weak signals. For some spheroid cells, the green signals totally disappeared (pointed by yellow arrows), suggesting that autophagosomes were degraded rapidly in lysosomes. Moreover, rapamycin could not stimulate a lot of green signals in spheroid cells as it did in adherent cells, suggesting autophagy was already activated in spheroid cells. These results suggested that autophagy was sustained activated in SKOV3 spheroid cells with most vacuoles becoming autolysosomes (red puncta). A2780 cells showed a similar pattern as shown in Figure S3(c).

### 3.2. Autophagy Promotes the Self-Renewal of Ovarian Cancer Spheroid Cells

**Figure 2:**
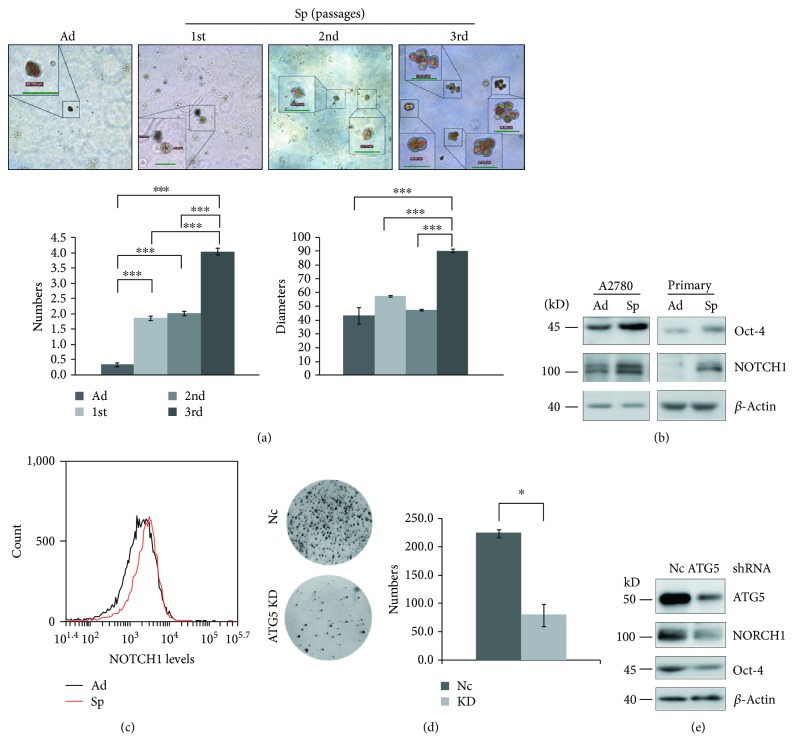
Autophagy promotes the self-renewal of ovarian cancer spheroid cells. (a) Soft agar colony formation assay of A2780 adherent and spheroid cells. Adherent and spheroid cells at different passages were seeded on the bottom layer and covered with the top layer. After 21 d, the numbers and diameters of the colonies were counted and measured (green scale bar, 50 *μ*m). (b) Western blot analysis of self-renewal markers in A2780 and primary adherent and spheroid cells. (c) Flow cytometry analysis of NOTCH1 levels in A2780 adherent and spheroid cells. The cells were trypsinized, fixed, stained with anti-NOTCH1 (1:50) antibody, and analyzed with flow cytometry. (d) Soft agar colony formation assay of Nc and ATG5 shRNA cell strains. 2 × 10^4^ of spheroid cells were cultured on soft agar for 28 d. The images were taken with a gel imaging system (Tanon), and the colonies were counted with ImageJ software (mean ± SEM, *n* = 3). (e) Western blot analysis of ATG5, NOTCH1, and Oct-4 in Nc and ATG5 shRNA A2780 spheroid cells.

Cancer stem cells can be enriched by serial passages of selection because of their self-renewal ability [31]. A2780 spheroid cells acquired the self-renewal ability as shown by forming more colonies than adherent cells on soft agar (Figure 2(a)). Moreover, the third passage of A2780 spheroid cells formed more and larger colonies than the second and first passages of spheroid cells (Figure 2(a)). Consistent with the self-renewal ability, Western blot and flow cytometry analyses showed that the protein levels of two self-renewal markers, Oct-4 and NOTCH1 [6, 32, 33], were upregulated in A2780 spheroid cells compared with adherent cells (Figures 2(b) and 2(c)). These results indicated that ovarian cancer spheroid cells become stem cell-like. Knockdown of ATG5 dramatically impaired the ability of A2780 spheroid cells to form colonies on soft agar (Figure 2(d)), indicating that autophagy is required for the self-renewal of ovarian cancer spheroid cells. Knockdown of ATG5 reduced NOTCH1 and Oct-4 protein levels in A2780 spheroid cells (Figure 2(e)).

### 3.3. Autophagy Is Critical for Ovarian Cancer Spheroid Cells to Maintain Quiescent State

**Figure 3:**
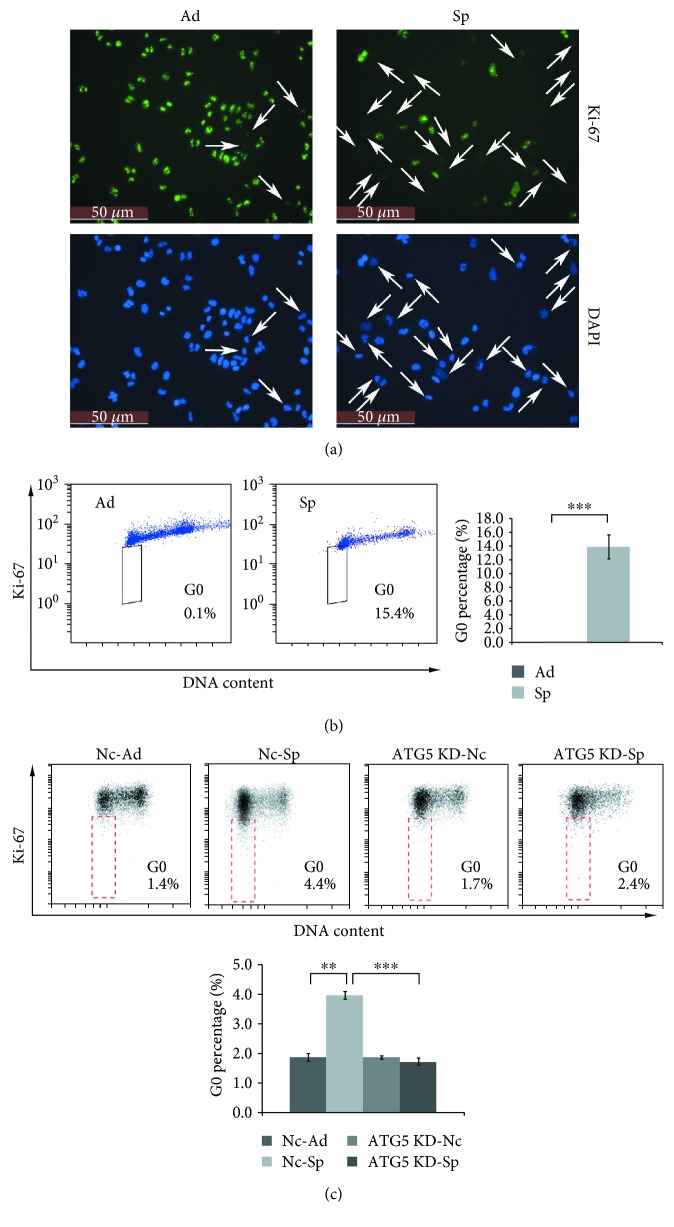
Autophagy is critical for ovarian cancer spheroid cells to enter quiescent state. (a) Immunostaining of Ki-67 in A2780 adherent and spheroid cells. A2780 cells were cultured under adherent or spheroid conditions for 48 h. The spheroid cells were trypsinized and seeded back to attach to the plate for 4 h; both the adherent and spheroid cells were fixed, stained with anti-Ki-67 antibody (green), counterstained with DAPI (blue), and observed with a fluorescence microscope. The experiment was repeated twice with similar results. (b) Flow cytometric analysis of G0 percentage of A2780 cells adherent and spheroid cells. A2780 cells were cultured under adherent or spheroid conditions for 48 h, trypsinized, fixed, and stained with propidium iodide and anti-Ki-67 antibody. Cells were subjected to flow cytometry analysis with Beckman Coulter FACS (mean ± SEM, *n* = 3). (c) Flow cytometric analysis of G0 percentage of Nc or ATG5 shRNA cells adherent and spheroid cells. Cells were subjected to flow cytometry analysis with BD FACS (mean ± SEM, *n* = 4).

Quiescent state (G0 phase) is essential to preserving the self-renewal capacity of stem cells. Cancer stem cells are thought to take advantage of quiescent state that supports normal stem cell behaviors [34–36]. Ki-67 can be detected among proliferating cells in G1, S, G2, and mitosis phases, but not in the G0 phase [37]. More quiescent cells were detected in A2780 spheroid cells compared with adherent cells (Figure 3(a), pointed out with white arrows). Flow cytometry analysis confirmed higher percentages of G0 cells existing in A2780 spheroid cells by simultaneously staining cells with propidium iodide and Ki-67 [25] (Figure 3(b)). Knockdown of ATG5 reduced the percentage of G0 cells in A2780 spheroid cells (Figure 3(c)). These results suggested that autophagy is required for ovarian cancer spheroid cells to enter quiescent state.

**Figure 4:**
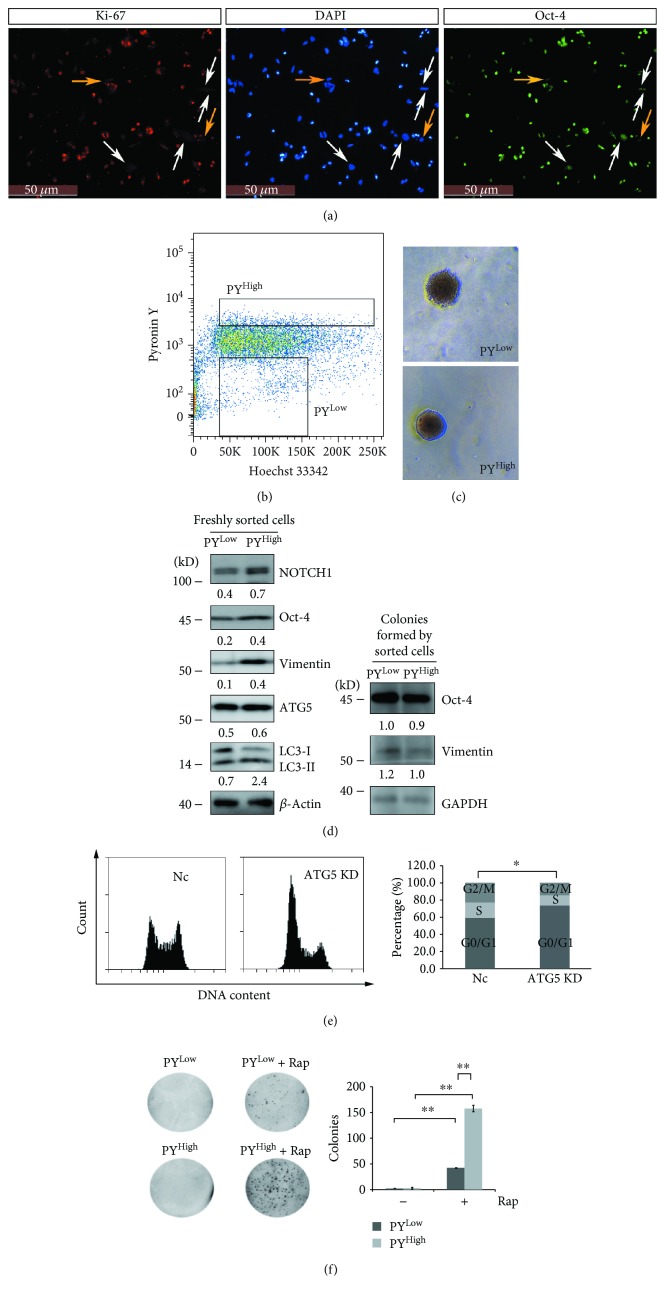
Autophagy promotes quiescent ovarian cancer spheroid cells to reenter the cell cycle. (a) Immunostaining of Oct-4 and Ki-67 in A2780 spheroid cells. Cells were cultured under spheroid culture condition for 48 h, trypsinized, and seeded back to attach to the plate for 4 h, fixed, stained with anti-Oct-4 antibody (green) and anti-Ki-67 antibody (red), counterstained with DAPI (blue), and observed with a fluorescence microscope. (b) Cell sorting of PY^Low^ and PY^High^ cells. A2780 spheroid cells were trypsinized, stained with Hoechst 33342 and pyronin Y, and sorted by flow cytometry. (c) Colony formation of sorted PY^Low^ and PY^High^ cells on soft agar. Sorted PY^Low^ and PY^High^ cells described in (b) were grown on soft agar for 28 d to form colonies. (d) Western blot analysis of the protein levels of Oct-4, NOTCH1, vimentin, ATG5, and LC3-I/II from extracts of freshly sorted PY^Low^ and PY^High^ cells or colonies formed by sorted PY^Low^ and PY^High^ cells. The colonies described in (c) were collected, resolved in SDS loading buffer, and subjected to Western blot analysis. The relative intensity of indicated proteins normalized to housekeeping protein was shown. LC3-II/LC3-I ratios were calculated. (e) Cell cycle analysis of Nc and ATG5 shRNA A2780 cell strains. The cells were trypsinized, fixed, digested with RNase A, stained with propidium iodide, and subjected to cell cytometry analysis (mean ± SEM, *n* = 3). (f) Colony formation of PY^Low^ and PY^High^ cells with or without rapamycin (Rap, 1 *μ*M) on soft agar. 1.5 × 10^4^ of PY^Low^ and PY^High^ cells were cultured on soft agar with or without Rap for 14 d. The images were taken with a gel imaging system, and the colonies were counted with ImageJ software (mean ± SEM, *n* = 2).

Quiescent stem cells have the ability to reenter the cell cycle, and the quiescence is a poised state regulated by different mechanisms [32]. We investigated whether autophagy can regulate quiescent ovarian cancer spheroid cells to reenter the cell cycle. Immunostaining showed that Oct-4 expression was relatively weak in Ki-67-negative cells compared with Ki-67-positive cells (Figure 4(a), pointed by white arrows). There were a few Ki-67-negative cells in which the Oct-4 signal almost disappeared (Figure 4(a), pointed by yellow arrows). These results suggested that Oct-4 may be categorized into the genes that are downregulated in the quiescent stem cells due to the low metabolic activity of the quiescent stem cells [32]. Quiescent cells can be sorted from other cells with the normal cell cycle by using Hoechst 3342/pyronin Y staining cell sorting, because quiescent cells maintain a low RNA content and can be separated from G1 cells [26]. We sorted quiescent spheroid cells (PY^Low^) from other cells (PY^High^) (Figure 4(b)). The sorted cells were vital because both sorted PY^Low^ and PY^High^ cells were able to form colonies on soft agar (Figure 4(c)). Western blot analysis was performed to confirm whether the Oct-4 level was lower in quiescent spheroid cells. As shown in Figure 4(d), in addition to Oct-4, the protein levels of NOTCH1 and vimentin were also lower in PY^Low^ compared with PY^High^ cells. After being grown on soft agar for 28 d, the protein levels of Oct-4 and vimentin were comparable in the colonies formed by the sorted PY^Low^ and PY^High^ cells (Figure 4(d)). These results suggested that quiescent cancer spheroid cells can reenter the cell cycle and the expression levels of downregulated genes (Oct-4 and Vimentin) can be restored after quiescent cancer spheroid cells reentered the cell cycle.

The LC3-II/LC3-I ratio was 3-fold higher in PY^High^ than PY^Low^ cells, but ATG5 was comparable between sorted PY^Low^ and PY^High^ cells (Figure 4(d)), indicating that autophagy flux might be lower in PY^Low^ cells, but the autophagy essential gene might be kept intact so that autophagy could be activated quickly. Indeed, knockdown of ATG5 induced A2780 cells arrested in G0/G1 (Figure 4(e)). To further investigate whether autophagy was able to regulate quiescent cancer spheroid cells to reenter the cell cycle, sorted PY^Low^ and PY^High^ cells were grown on soft agar with or without rapamycin for 14 d. As shown in Figure 4(f), rapamycin was able to promote both PY^low^ and PY^high^ cells to form more colonies on soft agar compared with PY^low^ and PY^high^ cells in the absence of rapamycin. The numbers of colonies were PY^high^ + rapamycin > PY^low^ + rapamycin > PY^high^ ≈ PY^low^. These results indicated that autophagy could promote quiescent cancer spheroid cells to reenter the cell cycle.

### 3.4. Autophagy and NRF2 Form a Positive Feedback Regulation Loop to Regulate the ROS Levels in Ovarian Cancer Spheroid Cells

**Figure 5:**
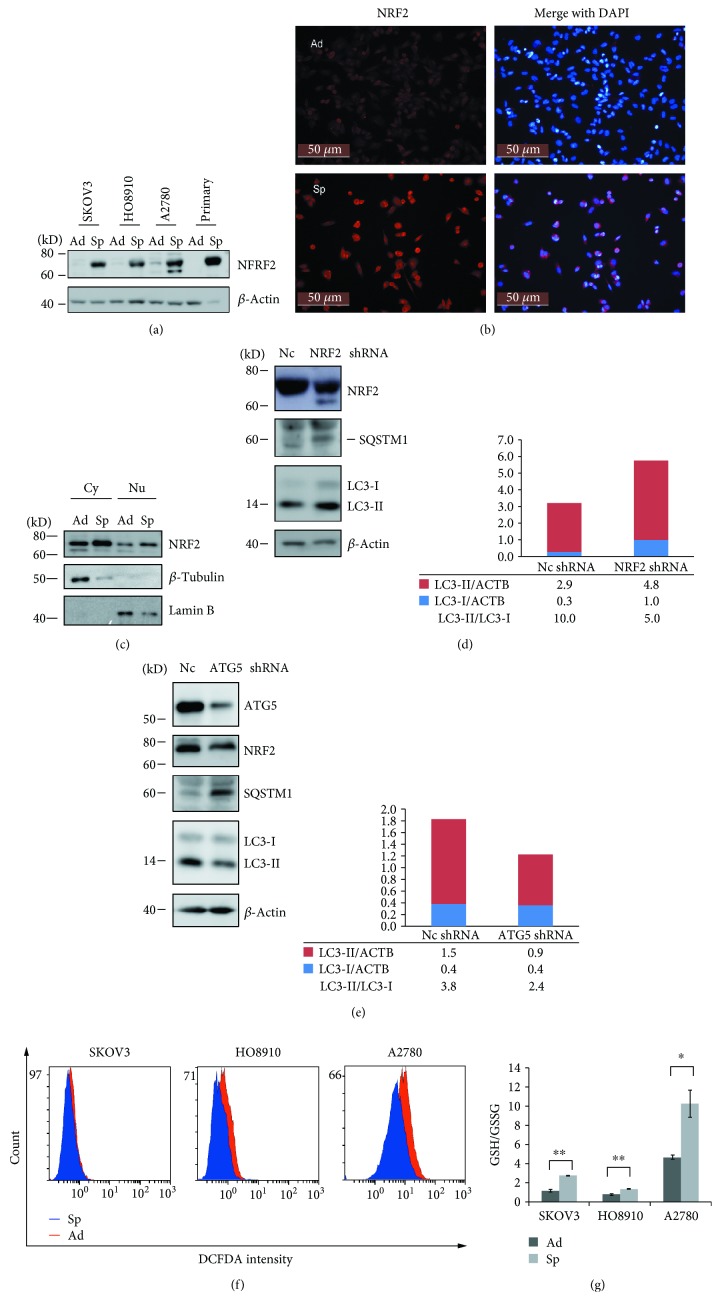
Autophagy and NRF2 form a positive feedback regulation loop to regulate the ROS levels in ovarian cancer spheroid cells. (a) Western blot analysis of NRF2 protein levels in adherent and spheroid ovarian cancer cells. (b) Immunostaining of NRF2 in adherent and spheroid A2780 cells. A2780 cells were cultured under adherent or spheroid culture conditions for 48 h. The spheroid cells were trypsinized and seeded back to attach to the plate for 4 h. Both adherent and spheroid cells were fixed and stained with NRF2 (red), counterstained with DAPI (blue), and observed with a fluorescence microscope. (c) Western blot analysis of cytoplasmic and nuclear NRF2 protein levels in A2780 adherent and spheroid cells. A2780 cells were cultured under adherent or spheroid conditions for 48 h; cytoplasmic and nuclear extracts were separated and blotted for NRF2 (cytoplasmic (Cy), nuclear (Nu)). (d–e) Western blot analysis of NRF2, ATG5, and autophagy markers in Nc, NRF2 shRNA, and ATG5 shRNA A2780 spheroid cells. The relative intensity of LC3-I or LC3-II normalized to *β*-actin (ACTB) was shown. The ratios of LC3-II/LC3-I were calculated. (f) ROS levels in ovarian cancer adherent and spheroid cells. Ovarian cancer cells were cultured under adherent or spheroid culture conditions for 48 h, trypsinized and stained with H2DCF, and measured with flow cytometry. (g) GSH/GSSH ratios in ovarian cancer adherent and spheroid cells. Ovarian cancer cells were cultured under adherent or spheroid conditions for 48 h, collected, and measured to calculate GSH/GSSG ratios (mean ± SEM, *n* = 3).

Nuclear factor E2-related factor 2 (NRF2) has been reported to be able to regulate the self-renewal and quiescence of haematopoietic stem cells, airway basal stem cells, and CSCs [38–40]. Our data showed that NRF2 levels were dramatically increased in spheroid cells of four ovarian cancer cell strains compared with adherent cells (Figure 5(a)). The increase in NRF2 protein levels was not due to enhanced transcription because NRF2 mRNA levels did not show difference between spheroid and adherent cells (Figure S4(a) and data not shown). Increased NRF2 levels were observed in both cytosol and nucleus of A2780 spheroid cells compared with adherent cells (Figures 5(b) and 5(c)). Knockdown of NRF2 by shRNA inhibited autophagy in A2780 spheroid cells as shown by enhanced p62/SQSTM1 level and reduced LC3-II/LC3-I ratio (Figure 5(d)). Blockage of autophagy by knockdown of ATG5 reduced the protein level of NRF2 in A2780 spheroid cells (Figure 5(e)). Thus, autophagy may form a positive feedback regulation loop with NRF2 in ovarian cancer spheroid cells.

According to the literatures, both autophagy and NRF2 are important to regulate intracellular antioxidant responses by controlling the expression of a lot of antioxidant genes [10, 41, 42]. Higher mRNA levels of many well-known NRF2 target genes [42–44] were detected in SKOV3 spheroid cells compared with adherent cells (Figure S4(b)). Knockdown of ATG5 enhanced ROS production which can be reduced by ROS scavenger N-acetyl cysteine (NAC) in A2780 cells (Figure S4(c)), indicating that autophagy can induce antioxidant response in ovarian cancer cells. These results suggested that autophagy and NRF2 may form a positive feedback loop to regulate intracellular ROS levels together. Compared with adherent cells, ROS levels and GSH/GSSG ratios (reduced to oxidized glutathione) were lower and higher in ovarian cancer spheroid cells, respectively (Figures 5(f) and 5(g)).

### 3.5. Autophagy Inhibitor Bafilomycin A1 Inhibits the Self-Renewal of Ovarian Cancer Spheroid Cells by Disturbing ROS Levels

**Figure 6:**
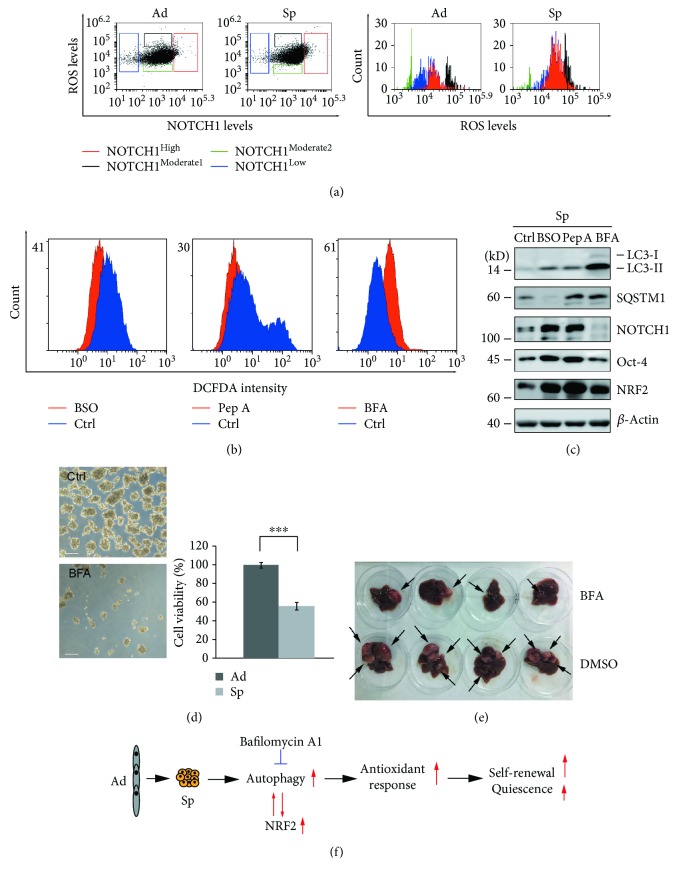
Bafilomycin A1 inhibits the self-renewal of ovarian cancer spheroid cells by perturbation of ROS levels. (a) The relationship between ROS levels and NOTCH1 levels. A2780 adherent or spheroid cells were double stained with H2DCF and anti-NOTCH1 antibodies. ROS levels and NOTCH1 levels were analyzed with flow cytometry. (b) ROS levels in A2780 spheroid cells treated with inhibitors. A2780 cells were cultured under spheroid culture conditions for 48 h in the presence or absence of the indicated inhibitors, stained with H2DCF, and measured with flow cytometry (buthionine sulfoximine, BSO, 800 *μ*M; pepstatin A, Pep A, 10 *μ*g/ml; and bafilomycin A1, BFA, 50 nM). (c) Western blot analysis of autophagy markers and self-renewal markers in A2780 spheroid cells treated with inhibitors. A2780 cells were cultured under spheroid culture condition for 48 h in the presence or absence of the indicated inhibitors. (d) The morphology and cell viability of A2780 spheroid cells were treated with or without bafilomycin A1. A2780 cells were cultured under spheroid culture condition for 48 h in the presence or absence of bafilomycin A1. Cells were trypsinized and stained with trypan blue to exclude dead cells. Scale bar: 50 *μ*m. The number of both live and dead cells was counted with a cell counter (Nexcelom Cellometer), and the percentages of cell viability were calculated (mean ± SEM, *n* = 3). (e) The size of human tumor xenografts treated with or without bafilomycin A1. Four mice per group were ip injected with 2 × 10^6^ A2789 spheroid cells. After 1 week, the mice were ip injected with DMSO or bafilomycin A1 (0.1 mg/kg) twice with 3 days of interval and sacrificed after 28 days. (f) Schematic diagram of how autophagy regulates the self-renewal of ovarian cancer spheroid cells with stem cell-like properties.

Decreased ROS levels are reported to be beneficial for the self-renewal of stem cells and cancer stem cells [12–14, 45]. We found that ROS levels were functionally related to NOTCH1 expression in ovarian spheroid cells. As shown in Figure 6(a), A2780 spheroid cells were double-stained with H2DCF and anti-NOTCH1 antibody. ROS levels and NOTCH1 levels were analyzed by flow cytometry. According to NOTCH1 levels, the cells were divided into four groups, NOTCH1^High^, NOTCH1^Moderate 1^, NOTCH1^Moderate 2^, and NOTCH1^Low^. The corresponding ROS levels were NOTCH1^Moderate 2^ < NOTCH1^Low^ < NOTCH1^High^ < NOTCH1^Moderate 1^. These data showed that relative low ROS levels favored NOTCH1 expression and there were more cells in spheroid cells that could be categorized into the NOTCH1^High^ group. However, when the decrease in ROS levels exceeded to a certain level, NOTCH1 expression was downregulated again (Figure 6(a)). These results indicated that perturbation of ROS levels may impair the self-renewal of ovarian spheroid cells. One GSH inhibitor, buthionine sulfoximine (BSO), which can activate autophagy [46], and two autophagy inhibitors pepstatin A and bafilomycin A1 were investigated for their ability to interfere ROS levels and the self-renewal of ovarian CSCs. All three inhibitors were capable of disturbing ROS levels in A2780 spheroid cells. ROS levels were increased by BSO and pepstatin A, but decreased by bafilomycin A1 (Figure 6(b)). Western blot analysis showed that NOTCH1 levels can be dramatically decreased by bafilomycin A1, but not by BSO or pepstatin A (Figure 6(c)). Moreover, bafilomycin A1 but not BSO or pepstatin A inhibited A2780 cells to form spheres and induced cell death (Figure 6(d) and data not shown). These results suggested that bafilomycin A1 inhibited the self-renewal of ovarian cancer spheroid cells by further decreasing ROS levels in spheroid cells. Bafilomycin A1 was able to inhibit the formation of tumor xenografts in nude mice as shown in Figure 6(e) (tumors were pointed by arrows) and Figure S5.

## 4. Discussion

Autophagy is sustainably activated in spheroid cells because the predominant forms of tfLC3 are autolysosomes in spheroid cells (Figure 1(c) and Figure S3(c)). Sustained activation of autophagy causes degradation of LC3-II; thus, an increase in LC3-II levels is not obvious but a decrease in LC-I levels is very clear in spheroid cells (Figure 1(a)).

p62/SQSTM1 is the autophagy substrate and can be degraded by autophagy [47]. However, we observe that the protein level of p62/SQSTM1 elevates when autophagy is activated in ovarian spheroid cells (Figure 1(a)). p62/SQSTM1 can function independent of autophagy [48] and be regulated by other factors [49]. Thus, the increased level of SQSTM1 will not change the overall autophagy activity in spheroid cells.

Ovarian cancer spheroid cells with stem cell-like properties are able to self-renew. Inhibition of autophagy by knockdown of ATG5 abolishes their self-renewal ability (Figure 2). The other critical character of stem cells is maintaining quiescence and reentering the cell cycle in response to environmental stimuli. Inhibition of autophagy by knockdown of ATG5 prevents ovarian cancer spheroid cells with stem cell-like properties to enter quiescent state (Figure 3). Quiescent ovarian cancer spheroid cells are able to form much more colonies on soft agar in the presence of rapamycin. ATG5 remains unchanged while other genes show lower levels in quiescent sorted cells, and knockdown of ATG5 induces the ovarian cancer cells arrested in G0/G (Figure 4). All these data suggest that quiescent ovarian cancer spheroid cells with stem-like properties may keep autophagy machinery so that autophagy may be quickly activated to create a suitable environment for quiescent spheroid cells to reenter the cell cycle. Indeed, autophagy is required to prevent the quiescent muscle stem cells from senescence [50]. It is proposed that the properties of stemness, including immortality, dormancy, chemo- or radioresistance, and epithelial-mesenchymal transition (EMT), are reversible at some time in a certain cancer cell population [2, 3, 51]. Decreased expression levels of Oct-4, NOTCH1, and vimentin in quiescent sorted ovarian cancer spheroid cells can be reversibly restored after the quiescent spheroid cells reenter the cell cycle and form new colonies (Figure 4(d)).

Autophagy forms a positive feedback loop with NRF2 to activate the antioxidant response in spheroid cells (Figure 5). The optimal ROS level, neither too high nor too low, facilitates the self-renewal marker, NOTCH1, to reach to the highest level. Pepstatin A and bafilomycin A1 can block autophagy by inhibiting lysosomal proteases and lysosome fusion, respectively. Moreover, bafilomycin A1 has been reported to be able to inhibit mitochondrial biogenesis dependent of autophagy in primary neurons [52]. Thus, pepstatin A treatment increases ROS levels in spheroid cells due to the inhibition of autophagy. But bafilomycin A1 can decrease ROS levels in spheroid cells as a consequence of inhibition of mitochondrial biogenesis. The ability to impair the self-renewal of ovarian cancer spheroid cells makes bafilomycin A1 a prominent candidate for ovarian cancer treatment.

## 5. Conclusion

In the present study, our data reveal that autophagy is essential to promoting self-renewal and maintaining the quiescence of ovarian cancer spheroid cells with stem cell-like properties. Autophagy forms a positive feedback regulation loop with NRF2 in ovarian cancer spheroid cells to activate antioxidant response together. Perturbation of the redox balance by bafilomycin A1 impairs the self-renewal of ovarian cancer spheroid cells and thus inhibits the tumor formation in vivo (Figure 6(f)).

## Data Availability

The microarray data used to support the findings of this study are included within the supplementary information file(s).

## Supplementary Materials

Supplementary 1 Figure S1: the morphology of ovarian cancer cells derived from three ovarian cancer cell lines under adherent or spheroid culture conditions.

Supplementary 2. Figure S2: identification of human primary ovarian cancer epithelial cells. (a) Immunostaining of keratin 18 in ovarian cancer epithelial cells and cancer-associated fibroblasts. Cells were stained with keratin 18 (red) and counterstained with DAPI (blue). (b) The morphology of primary ovarian cancer epithelial cells under adherent or spheroid culture conditions.

Supplementary 3 Figure S3: autophagy is activated in ovarian cancer spheroid cells. (a) Heat map of autophagy essential genes differentially expressed between SKOV3 adherent and spheroid cells. (b) Western blot analysis of LC3 and SQSTM1 in A2780 spheroid cells treated with bafilomycin (BFA, 50 nM) or chloroquine (CQ, 50 *μ*M). The relative intensity of SQSTM1 was normalized to the housekeeping protein. The quantification of LC3 was shown by the ratio of LC3-II/LC3-I. (c) Fluorescence signals of tfLC3 in A2780 adherent and spheroid cells treated with or without rapamycin (Rap, 10 *μ*M).

Supplementary 4 Figure S4: autophagy and NRF2 activate the antioxidant response in ovarian cancer spheroid cells. (a) qRT-PCR analysis of NRF2 in negative control (Nc) and NRF2 shRNA A2780 cells (mean ± SEM, *n* = 3). (b) Heat map of NRF2 targets differentially expressed between SKOV3 adherent and spheroid cells. (c) ROS levels in A2780 cells silenced with ATG5 siRNA. Adherent A2780 cells were transiently transfected with Nc or ATG5 siRNA for 24 h, incubated with or without NAC (5 mg/ml) for 2 h, and further cultured in complete media for another 24 h. Cells were stained with H2DCF (20 *μ*M) at 37°C for 15 min. The fluorescence intensity was determined by flow cytometry. The knockdown efficiency of ATG5 was analyzed by Western blot. The relative intensity of ATG5 normalized to housekeeping protein was shown.

Supplementary 5 Figure S5: bafilomycin A1 was able to inhibit the formation of tumor xenografts in nude mice. (a) Phase or fluorescent images of the morphology of A2780-mCherry-Luc strain. (b) In vivo imaging. The nude mice were ip injected with 2 × 10^6^ A2780-mCherry-Luc spheroid cells. After 1 week, the mice were ip injected with DMSO or bafilomycin A1. After 28 days, the mice were ip injected with D-luciferin and scanned with imaging system (Roper Scientific).

Supplementary 6 Microarray analysis: RNA samples from SKOV3 adherent and spheroid cells were prepared for the microarray analysis. The microarray was performed by Shanghai Biochip Co., Ltd. (China) with Affymetrix GeneChip arrays. The gene expression data were provided as supplementary material. The heat map of gene differentiation was generated using the method described by Rubin et al. [21]. Briefly, a heat map of the median-centered FPKM (fragments per kilobase of exon per million fragments mapped) values corresponding to the transcripts in samples was generated using the heatmap.2 function of the gplots R package. Reverse transcription and q-PCR. Cells were lysed with RNAiso reagent (TaKaRa, Japan). Total RNA was extracted, and 1 *μ*g of RNA was reverse-transcribed with M-MLV Reverse Transcriptase (Promega, USA) and subjected to quantitative PCR (q-PCR). The primer sequences are as follows: p62/SQSTM1 forward 5′-CTGAAGAACGTTGGGGAGAG-3′, reverse 5′-GCTCTTCTCCTCTGTGCTGG-3′; NRF2 forward 5′-ATCATGATGGACTTGGAGCTG-3′, reverse 5′-GCTCATACTCTTTCCGTCGC-3′; And 18s RNA forward 5′-CGTTGATTAAGTCCCTGCCCTT-3′, reverse 5′-TCAAGTTCGACCGTCTTCTCAG-3′. Animal imaging assay. For establishing shRNA cell lines, A2780 cells were transfected with pGPU6/shRNA constructs and selected by complete medium containing 100 *μ*g/ml neomycin. For establishing A2780 stably expressing mCherry and luciferase (A2780-mCherry-Luc), HEK 293T cells were transfected with pLVX-mCherry-C1-Fluc, pMD2.G, and psPAX2 to produce lentivirus to transduce A2780 cells in the presence of polybrene. The transduced A2780 cells were selected by complete medium containing 10 *μ*g/ml puromycin. The mice were ip injected with 2 × 106 A2780-mCherry-Luc spheroid cells. After 1 week, the mice were ip injected with DMSO or bafilomycin A1 (0.1 mg/kg) twice with 3 days of interval. After 28 days, the mice were ip injected with D-luciferin (15 mg/kg) and scanned with an imaging system (Roper Scientific).
