# Supplementary figures and images for: Autophagy Is Indispensable for the Self-Renewal and Quiescence of Ovarian Cancer Spheroid Cells with Stem Cell-Like Properties
Source: Oxid Med Cell Longev. 2018 Sep 17;2018:7010472. doi: 10.1155/2018/7010472 (PMC6167563; doi:10.1155/2018/7010472)

Ad

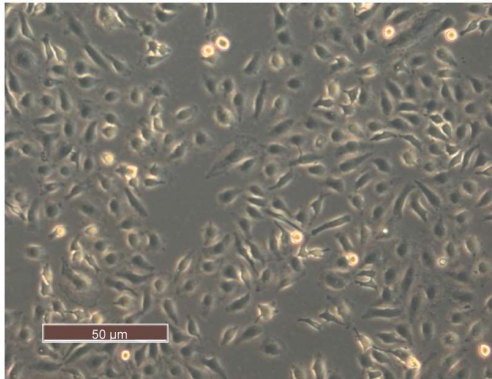

Sp

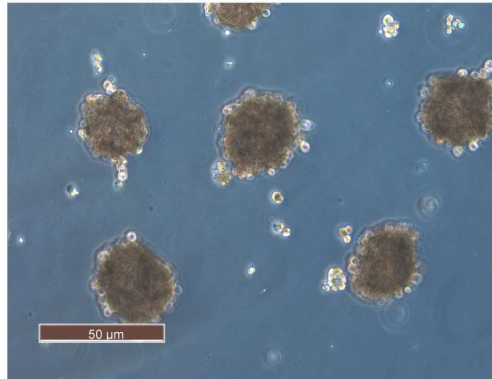

SKOV3

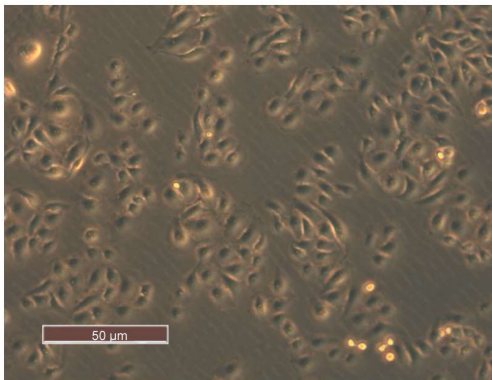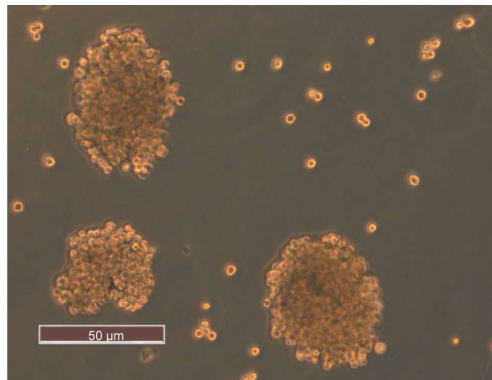

HO8910

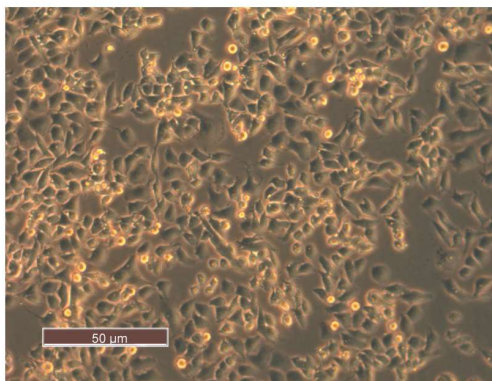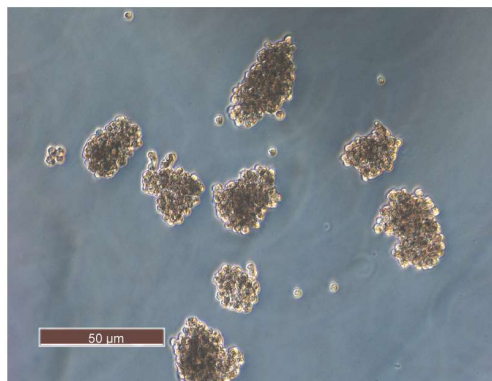

A2780

Supplement: Supplementary 1 — Figure S1: the morphology of ovarian cancer cells derived from three ovarian cancer cell lines under adherent or spheroid culture conditions. [file 7010472.f1.pdf]

a

Epithelia  
cells

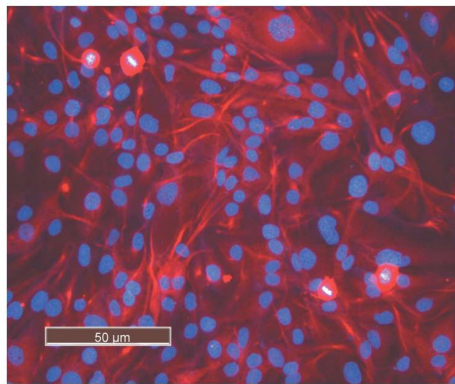

b

Ad

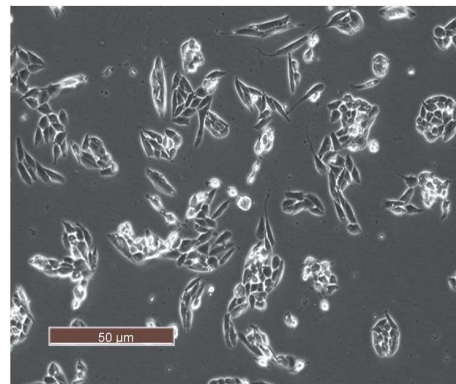

Fibroblasts

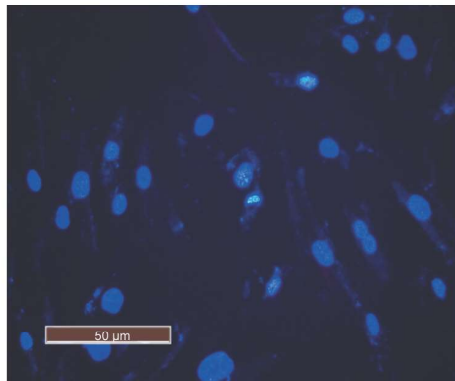

Sp

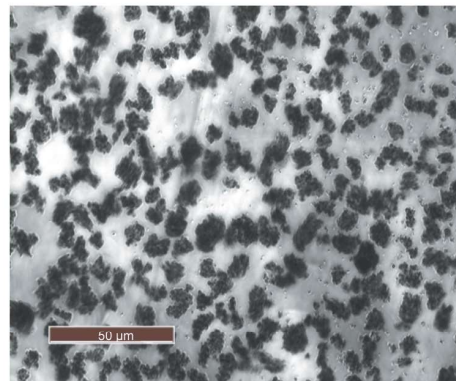

Supplement: Supplementary 2. — Figure S2: identification of human primary ovarian cancer epithelial cells. (a) Immunostaining of keratin 18 in ovarian cancer epithelial cells and cancer-associated fibroblasts. Cells were stained with keratin 18 (red) and counterstained with DAPI (blue). (b) The morphology of primary ovarian cancer epithelial cells under adherent or spheroid culture conditions. [file 7010472.f2.pdf]

Figure S3

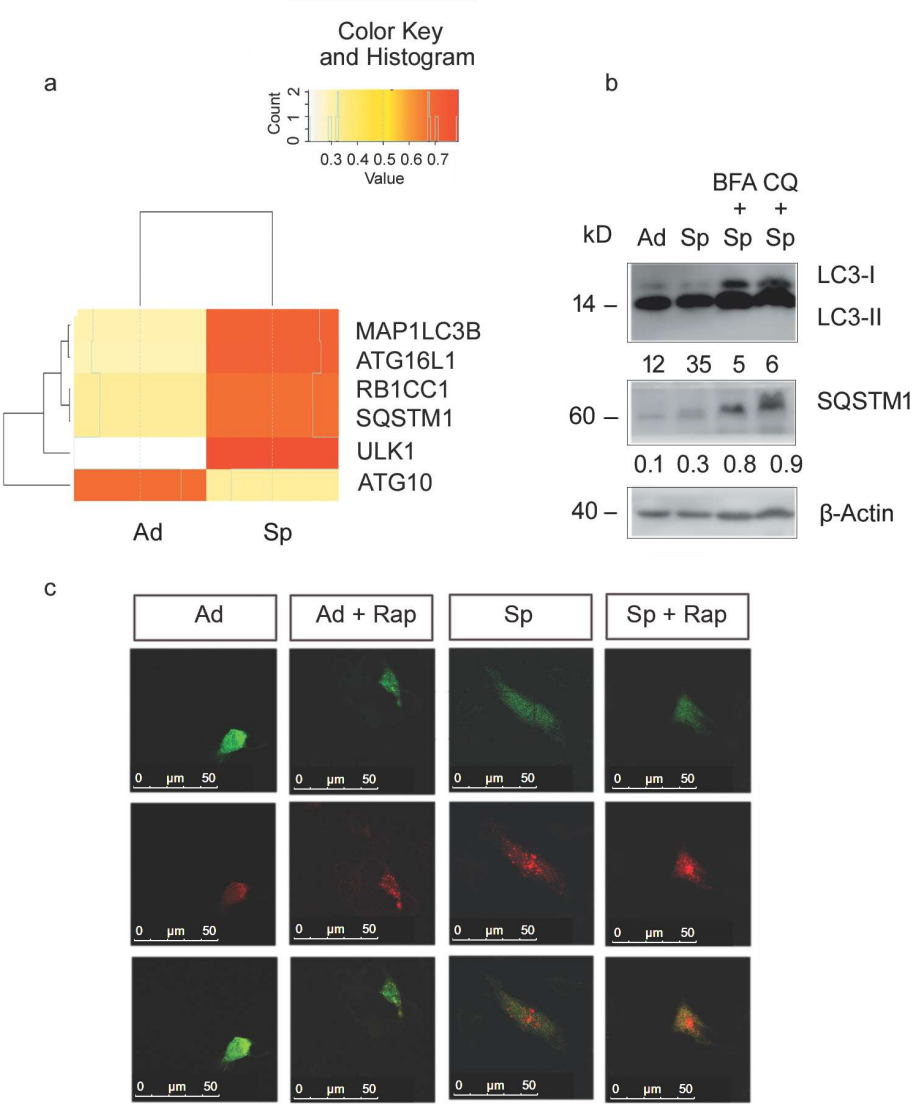

Supplement: Supplementary 3 — Figure S3: autophagy is activated in ovarian cancer spheroid cells. (a) Heat map of autophagy essential genes differentially expressed between SKOV3 adherent and spheroid cells. (b) Western blot analysis of LC3 and SQSTM1 in A2780 spheroid cells treated with bafilomycin (BFA, 50 nM) or chloroquine (CQ, 50 μM). The relative intensity of SQSTM1 was normalized to the housekeeping protein. The quantification of LC3 was shown by the ratio of LC3-II/LC3-I. (c) Fluorescence signals of tfLC3 in A2780 adherent and spheroid cells treated with or without rapamycin (Rap, 10 μM). [file 7010472.f3.pdf]

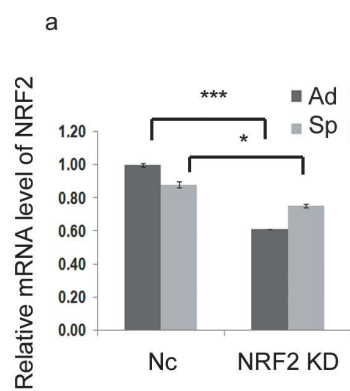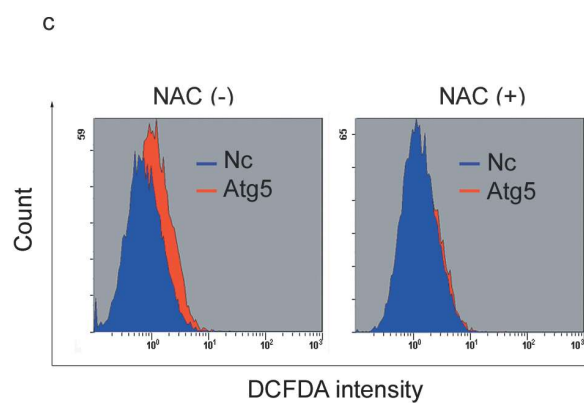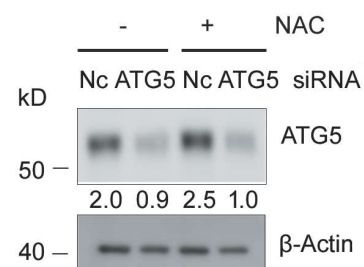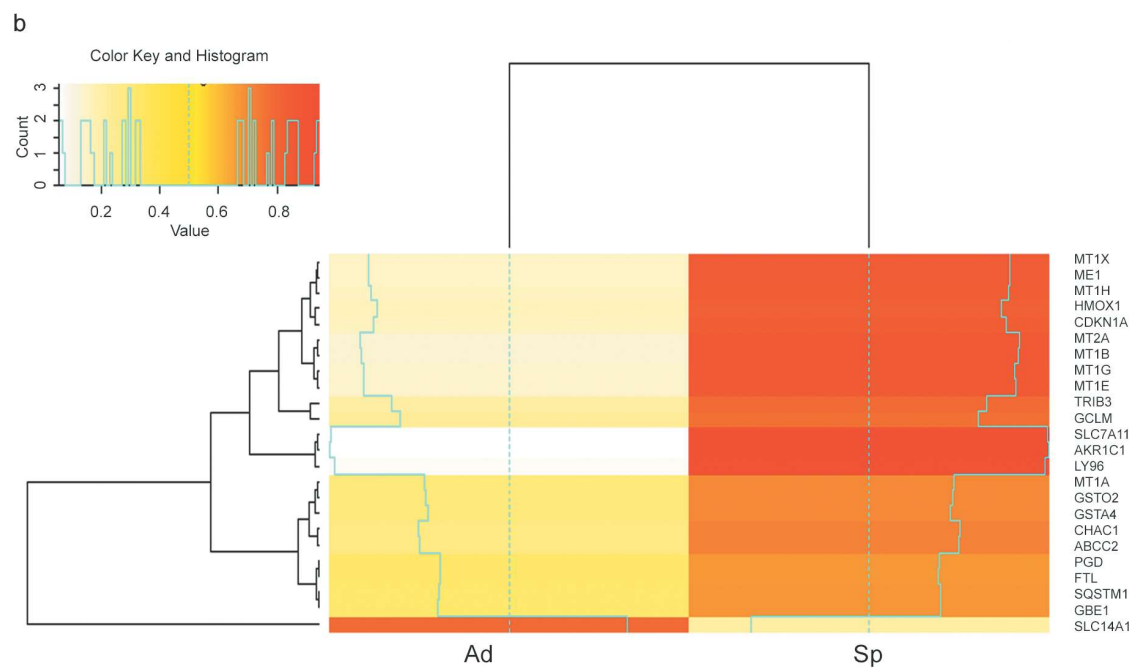

Supplement: Supplementary 4 — Figure S4: autophagy and NRF2 activate the antioxidant response in ovarian cancer spheroid cells. (a) qRT-PCR analysis of NRF2 in negative control (Nc) and NRF2 shRNA A2780 cells (mean ± SEM, n = 3). (b) Heat map of NRF2 targets differentially expressed between SKOV3 adherent and spheroid cells. (c) ROS levels in A2780 cells silenced with ATG5 siRNA. Adherent A2780 cells were transiently transfected with Nc or ATG5 siRNA for 24 h, incubated with or without NAC (5 mg/ml) for 2 h, and further cultured in complete media for another 24 h. Cells were stained with H2DCF (20 μM) at 37°C for 15 min. The fluorescence intensity was determined by flow cytometry. The knockdown efficiency of ATG5 was analyzed by Western blot. The relative intensity of ATG5 normalized to housekeeping protein was shown. [file 7010472.f4.pdf]

Figure S5

a

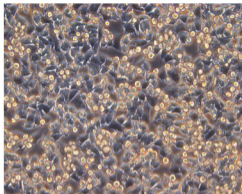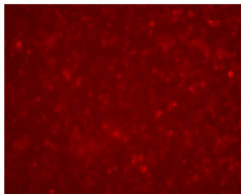

b

Ctrl

BFA

Ctrl

BFA

Ctrl

BFA

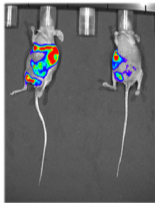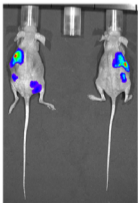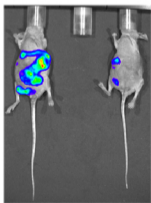

Supplement: Supplementary 5 — Figure S5: bafilomycin A1 was able to inhibit the formation of tumor xenografts in nude mice. (a) Phase or fluorescent images of the morphology of A2780-mCherry-Luc strain. (b) In vivo imaging. The nude mice were ip injected with 2 × 10^6^ A2780-mCherry-Luc spheroid cells. After 1 week, the mice were ip injected with DMSO or bafilomycin A1. After 28 days, the mice were ip injected with D-luciferin and scanned with imaging system (Roper Scientific). [file 7010472.f5.pdf]
